# Abnormal expression and function of the E-cadherin–catenin complex in gastric carcinoma cell lines

**DOI:** 10.1038/sj.bjc.6690358

**Published:** 1999-05-01

**Authors:** A U Jawhari, M Noda, M J G Farthing, M Pignatelli

**Affiliations:** Digestive Diseases Research Centre, St Bartholomew's and the Royal London School of Medicine and Dentistry, Turner Road, Whitechapel, London E1 2AD, UK; Imperial College School of Medicine, Division of Investigative Science, Department of Histopathology, Hammersmith Hospital, Du Cane Road, London W12 0NN, UK; Third Department of Internal Medicine, Kyoto Prefectural University of Medicine, Kyoto 602, Japan

**Keywords:** cadherin, catenin, gastric carcinoma, cell adhesion

## Abstract

Dysfunction of the cadherin–catenin complex, a key component of adherens junctions, is thought to confer invasive potential to cells. The aim of this study is to examine the expression and function of the E-cadherin/catenin complex in gastric carcinoma cell lines. Expression of E-cadherin, α, β and γ-catenin and p120*^ctn^*, and of the adenomatous polyposis coli protein (APC), together with function of the cadherin–catenin complex was examined in a panel of gastric carcinoma cell lines, using immunocytochemistry, Western blotting and a cell–cell aggregation assay. Protein interactions were examined by sequential immunoprecipitation and immunoblotting with antibodies to E-cadherin, α, β and γ-catenin, p120*^ctn^* and APC. Abnormalities of E-cadherin, α- and β-catenin expression, were associated with disturbance of E-cadherin–catenin complex composition, loss of membranous localization and loss of calcium-dependent aggregation in six gastric carcinoma cell lines. APC protein expression and interaction with β-catenin was preserved in five cell lines. We demonstrate frequent abnormalities of expression and function of E-cadherin and catenins, and associated disturbance of E-cadherin-mediated intercellular adhesion in gastric carcinoma cell lines. These findings support the tumour suppressor role of the E-cadherin and its contribution to the development and progression of the neoplastic phenotype in gastric carcinoma. © 1999 Cancer Research Campaign


*British Journal of Cancer* (1999) **80**(3/4), 322–330
© 1999 Cancer Research Campaign
Article no. bjoc.1998.0358

### Abnormal expression and function of the E-cadherin–catenin complex in gastric carcinoma cell lines

**AU Jawhari[1], M Noda[2,3], MJG Farthing[1] and M Pignatelli[2]**

[1]Digestive Diseases Research Centre, St Bartholomew's and the Royal London School of Medicine and Dentistry, Turner Road, Whitechapel, London E1 2AD, UK; [2]Imperial College School of Medicine, Division of Investigative Science, Department of Histopathology, Hammersmith Hospital, Du Cane Road, London W12 0NN, UK; [3]Third Department of Internal Medicine, Kyoto Prefectural University of Medicine, Kyoto 602, Japan

**Summary** Dysfunction of the cadherin–catenin complex, a key component of adherens junctions, is thought to confer invasive potential to cells. The aim of this study is to examine the expression and function of the E-cadherin/catenin complex in gastric carcinoma cell lines. Expression of E-cadherin, α, β and γ-catenin and p120*ctn*, and of the adenomatous polyposis coli protein (APC), together with function of the cadherin–catenin complex was examined in a panel of gastric carcinoma cell lines, using immunocytochemistry, Western blotting and a cell–cell aggregation assay. Protein interactions were examined by sequential immunoprecipitation and immunoblotting with antibodies to E-cadherin, α, β and γ-catenin, p120*ctn* and APC. Abnormalities of E-cadherin, α- and β-catenin expression, were associated with disturbance of E-cadherin–catenin complex composition, loss of membranous localization and loss of calcium-dependent aggregation in six gastric carcinoma cell lines. APC protein expression and interaction with β-catenin was preserved in five cell lines. We demonstrate frequent abnormalities of expression and function of E-cadherin and catenins, and associated disturbance of E-cadherin-mediated intercellular adhesion in gastric carcinoma cell lines. These findings support the tumour suppressor role of the E-cadherin and its contribution to the development and progression of the neoplastic phenotype in gastric carcinoma.

**Keywords:** cadherin; catenin; gastric carcinoma; cell adhesion

E-cadherin is a calcium-dependent cell–cell adhesion molecule, which forms the key functional component of adherens junctions of all epithelial cells. It interacts with E-cadherin molecules on adjacent epithelial cells forming an adhesive structure, which has been likened to an intercellular zipper (Shapiro et al, 1995). It plays a key role in the establishment and maintenance of intercellular adhesion, cell polarity and tissue architecture (Takeichi, 1991). Translation of intercellular contact signals into cellular organization is thought to be mediated by the catenins, which are key regulatory molecules in this mechanism. α-catenin (102 kDa), β-catenin (94 kDa) and γ-catenin (82 kDa), are membrane undercoat proteins which, through a series of interactions, link the cytoplasmic carboxy-terminal tail of E-cadherin to the actin cytoskeleton (Nagafuchi and Takeichi, 1989).

α-catenin is homologous to Vinculin, a protein localized to focal contacts and adherens junctions, and known to play a role in linking these junctions to the actin cytoskeleton (Geiger et al, 1980). β-catenin shows homology to the product of the *Drosophila* segment polarity gene *armadillo*, and is an important component of the *Wingless-Wnt* signalling cascade (Gumbiner, 1995; Peifer, 1995). Its role in signal transduction is independent of its adhesive function or role in transcriptional regulation (Behrens et al, 1996). β-catenin also interacts with the epidermal growth factor receptor (EGFR) (Hoschuetzky, 1994), and the proto-ongcogene c-erbB2 (Ochiai et al, 1994), both members of the type I growth factor receptor family, which have intrinsic tyrosine kinase activity. Both EGFR and c-erbB2 genes are amplified in a number of tumours, including gastric cancer (Lemoine et al, 1991). Phosphorylation of β-catenin, possibly through interaction with EGFR and c-erbB2, is thought to induce a disassembly of the E-cadherin–catenin complex from the actin filament network thus disruption of cell adhesion and may in turn potentiate the neoplastic process (Shibamoto et al, 1994).

The demonstration of an APC–β-catenin interaction raised much speculation as to the role of APC in regulating signal transduction through the β-catenin interaction with the cytoskeleton, thereby regulating some aspect of cell adhesion or cell growth (Rubinfeld et al, 1993; Su et al, 1993). APC has been shown to be important in regulating cytoplasmic β-catenin levels (Munemitsu et al, 1995). The APC–β-catenin complex is translocated into the nucleus where it interacts with the nuclear transcription factors LEF-1 and hTcf-4 (Korinek et al, 1997), resulting in transcriptional activation. Truncation of the carboxy-terminal domain of the wild-type APC protein, in tumours containing *APC* gene mutations (Munemitsu et al, 1995) results in accumulation of cytoplasmic β-catenin, which is thought to contribute to the inappropriate activation of target genes by the β-catenin–LEF–TCF complex.

γ-catenin bears homology to β-catenin and is identical to desmosomal plakoglobin. p120*ctn* is the most recently discovered member of the catenin family. It is phosphorylated by pp60$^{v\text{-}src}$, a tyrosine kinase known to be associated with the adherens junction (Volberg et al, 1992), and which induces disruption of adherens junctions and epithelial cell transformation when overexpressed.

*Received 1 June 1998*
*Revised 4 November 1998*
*Accepted 6 November 1998*

*Correspondence to:* M Pignatelli

$p120^{ctn}$ is also tyrosine phosphorylated in response to stimulation by mitogenic stimuli, including epidermal growth factor (EGF) and colony-stimulating factor 1 (CSF1; Downing and Reynolds, 1991). These observations have suggested a role for $p120^{ctn}$ in cell transformation and ligand-induced signalling. The existence of multiple $p120^{ctn}$ isoforms is thought to represent a means for modulating E-cadherin function, by selective expression (Staddon et al, 1995).

A number of lines of evidence have implicated E-cadherin as a tumour suppressor. Deletions or mutations of E-cadherin result in disruption of adherens junction function and loss of calcium-dependent cell–cell adhesion (Nagafuchi and Takeichi, 1988). The chromosome 16q region and containing the E-cadherin gene is affected by loss of heterozygosity in breast and prostate cancers (Sato et al, 1990; Bergerheim et al, 1991; Carter et al, 1991). Functional disturbance of the adherens junction may, however, also result from defects of catenin structure and function (Hirano et al, 1992; Kawanishi et al, 1995). In vivo studies have demonstrated reduced immunoreactivity of E-cadherin, α- and β-catenin in a wide variety of tumours (Shiozaki et al, 1991, 1994), including breast (Oka et al, 1993), gastric (Shimoyama and Hirohashi, 1991), (Matsuura et al, 1992; Becker et al, 1993, 1994), colorectal (Nigam et al, 1993), pancreatic (Pignatelli et al, 1994) and urogenital cancers (Umbas et al, 1992). An inverse relationship to tumour grade and stage was noted (Oka et al, 1993; Pignatelli et al, 1994) and in some cases a correlation with reduced survival (Umbas et al, 1992; Bringuier et al, 1993; Jawhari et al, 1997).

Transformation from a normal epithelial cell to a malignant cell is thought to be a multistep process and results from accumulation of multiple gene abnormalities. A number of genetic and chromosomal defects have been described in gastric carcinoma, but no unifying hypothesis for the development of this tumour has emerged (Tahara, 1995). Somatic E-cadherin mutations have been described in up to 50% of diffuse-type gastric tumours and 14% of mixed type tumours, but not intestinal tumours (Becker et al, 1993). A recent study reported a germline E-cadherin mutation leading to a truncated gene product, responsible for early onset, poorly differentiated gastric cancer in a large kindred from New Zealand (Guilford et al, 1998). Inactivating E-cadherin mutations were also identified in other gastric cancer families as well as somatic mutations in a number of sporadic gastric cancers, suggesting an important role for E-cadherin in the pathogenesis of gastric cancer.

To elucidate the role of the E-cadherin–catenin complex in gastric carcinoma, we examined expression and function of complex components in a panel of gastric carcinoma cell lines. Our results demonstrate evidence of aberrant expression and function of this complex in four of these cell lines.

### MATERIALS AND METHODS

### Cell lines

Kato 3, AGS, HT29 and MDCK, were obtained from the European tissue culture collection. MKN7 and MKN45 were obtained from Riken Cell Bank, Ibaraki, Japan, by kind permission of the originator, Dr Teiichi Motoyama. HSC39 and HSC40a were kindly donated by the originator, Dr Kazuyoshi Yangihara.

Kato III is a cell line derived from a metastatic pleural effusion secondary to signet ring carcinoma of the stomach. MKN45 is derived from a moderate to poorly differentiated intestinal type gastric carcinoma, while MKN7 is derived from a well-differentiated, intestinal-type gastric carcinoma. HSC39 was derived from malignant ascites arising secondary to a signet ring carcinoma, while HSC40a was established from the same original tumour after xenotransplantation of ascitic tumour cells into an athymic BALB/c nude mouse.

### Growth and maintenance of cells

Cells lines were maintained in Dulbecco's modified Eagle's medium (DMEM; Gibco BRL) supplemented with 10% heat inactivated, mycoplasma screened fetal calf serum (Gibco). The cells were cultured at 37°C in a humidified atmosphere containing 10% carbon dioxide. Cells were tested for mycoplasma at 3-monthly intervals.

### Antibodies

Mouse monoclonal IgG antibodies to E-cadherin, α-, β- and γ-catenin and $p120^{ctn}$, were purchased from Affinity Research Products Ltd, Exeter, UK. Two antibodies to the EGFR were used, EGFR1 a mouse monoclonal antibody, and 12E a rabbit polyclonal antibody, were kindly donated by Professor W Gullick (Imperial Cancer Research Fund (ICRF), London) (Wilding et al, 1996). The APC antibody, Ab28, was kindly donated by Sir Walter Bodmer (ICRF) (Efstathiou et al, 1998).

### Immunofluoresence staining of cultured cells

Cells were grown on multiwell slides (Hendley, Essex, UK), and fixed in 50:50 mixture of methanol and acetone at − 20 C for 20 min. Incubation with primary antibodies was performed overnight at 4°C. After washing in phosphate-buffered saline (PBS), the slides were incubated with biotinylated species-specific IgG for 30 min (Dako Ltd, High Wycombe, UK). After further washes in PBS, the slides were incubated in fluorescence isothiocyanate (FITC)-labelled avidin (Vector Laboratories, Peterborough, UK) for 10 min. Slides were mounted in Vecta shield (Vector Laboratories). For negative controls, primary antibody was replaced with mouse IgG.

### Cell aggregation assay

The assay was performed as described by Takeichi (1977) with modifications. Single cell suspensions were prepared by incubation in non-enzymatic dissociation media (Sigma-Aldrich Company Ltd., Poole, UK) for 10 min at 37°C. The dissociated cells were washed twice in calcium- and magnesium-free medium and suspended in either calcium- and magnesium-free medium, or normal calcium containing medium at a concentration of $1 \times 10^6$ cell $ml^{-1}$. The cells were then transferred to 50-ml Falcon tubes, preincubated with bovine serum albumin to reduce cells adhering to the plastic. The cell suspension was incubated on a gyratory shaker at 37°C. The number of single cells were counted at time 0 and at 15 min intervals during the experiment using a size-gated Coulter counter (Coulter Electronics, UK). Cell aggregation is represented by the aggregation index, $N_t/N_0$, which represents the ratio of single particles at time t of incubation to the initial single particle number at time 0. The aggregation index is plotted on the *y*-axis against time on the *x*-axis.

© Cancer Research Campaign 1999

### Immunoprecipitation

Cell extracts were prepared by lysis on ice, with 0.5% Triton X-100 buffer [50 mM sodium chloride (NaCl), 10 mM Pipes, 3 mol magnesium chloride ($MgCl_2$), 300 mM sucrose] with added protease inhibitors, as described above. Preclearing of cell extracts was performed using mouse IgG (2 μg ml$^{-1}$ of cell extract), to reduce non-specific binding. Protein G sepharose beads (50 μl) (Pharmacia Biotech, St Albans, UK) were prewashed in lysis buffer, and incubated with cell extract in the presence of mouse IgG (2 μl), for 1 h at 4°C on a rotator. The slurry was then centrifuged, and the cell extract transferred to a new tube containing protein G (50 μl), prewashed and incubated in the presence of antibody (2 μl), for 2 h at 4°C on a rotator. The samples were sequentially washed with high stringency buffer (15 mM Tris–HCl, 5 mM EDTA, 2.5 mM EGTA, 1% Triton X-100, 1% sodium deoxycholate, 0.1% sodium dodecyl sulphate (SDS), 120 mM NaCl, 25 mM potassium chloride), high salt buffer (15 mM Tris–HCl, 5 M EDTA, 2.5 mM EGTA, 1% Triton X-100, 1% sodium deoxycholate, 1% SDS, 1 M NaCl) and low salt buffer (15 mM Tris–HC1, 5 mM EDTA). Wash buffer was aspirated with a needle and the precipitated proteins eluted from the beads under reducing conditions by boiling in Laemmli sample buffer for 5 min. Composition of the eluted protein complexes was analysed by SDS-polyacrylamide gel electrophoresis (SDS-PAGE) and Western blotting. To confirm immunoprecipitation results and ascertain that lysis and wash conditions did not influence reported protein–protein interactions, cell lysis was also performed using the milder NP-40 buffer (50 mM Tris pH 7.5, 100 mM NaCl, 0.5% NP-40, 0.2 mM $Na_3VO_4$, 50 mM NaF). Protein G beads were subsequently washed gently using this same buffer (data not shown).

### SDS-PAGE and Western blotting

Cell extracts were prepared using Laemmli sample buffer (715 mM 2-β-mercaptoethanol, 10% glycerol, 2% SDS, 40 mM Tris pH 6.8, 1 mM EDTA; Laemmli, 1970), with a protease inhibitor cocktail (aprotinin 0.1–2 μg ml$^{-1}$, leupeptin 0.5–2 μg ml$^{-1}$, PMSF 20–100 μg ml$^{-1}$, trypsin-chymotrypsin inhibitor 10 μg ml$^{-1}$, TPCK 100 μg ml$^{-1}$ (all from Sigma-Aldrich Company Ltd, Poole, UK). Sample protein concentration was determined by BioRad protein assay (BioRad, Hemel Hempstead, UK), using spectrophotometry. SDS-PAGE was performed under reducing conditions using an 8% acrylamide resolving gel, pH 8.8, overlaid with a 4.5% stacking gel pH 6.8. The separated proteins were transferred to nitrocellulose (Millipore, UK), using BioRad apparatus. Visualization of protein bands was achieved by incubation in primary antibody overnight followed by extensive washing, and then in horseradish peroxidase-labelled, secondary species-specific antibody (Dako Ltd, High Wycombe, UK). The membrane was developed in ECL chemiluminescent reagent (Amersham Life Science, Slough, UK) and exposed to X-ray film.

### RESULTS

### Functional assessment of cadherin-mediated intercellular adhesion in six gastric carcinoma cell lines

Cadherin function is reflected by the cell phenotype when grown in culture and can be quantitatively measured using a cell–cell aggregation assay. HT29 is a colon carcinoma cell line, which forms a typical polygonal cell sheet in culture, characteristic of epithelial cells. Previous characterization of the cadherin–catenin complex in HT29 has established this to be intact. It was therefore used as control.

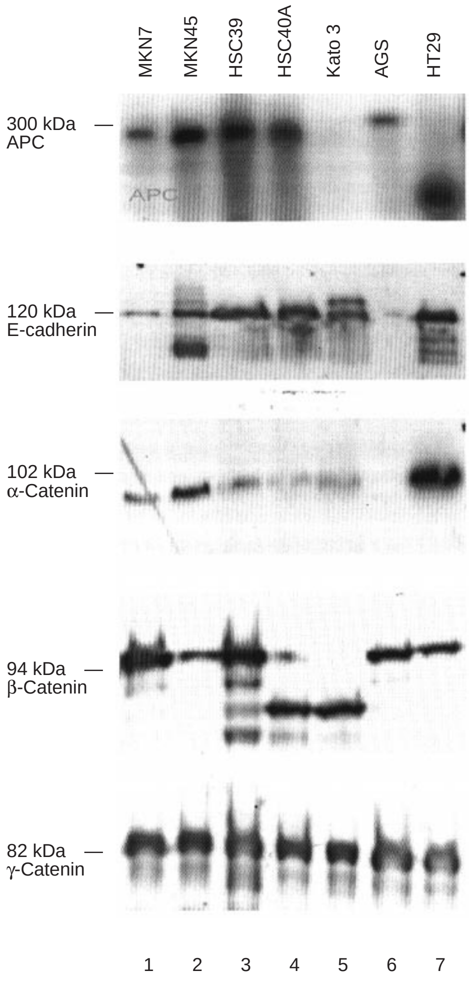

**Figure 1** Western blotting results for APC, E-cadherin, α-, β- and γ-catenin in five gastric carcinoma cell lines. The results of Western blotting for APC, E-cadherin, α-, β- and γ-catenin are shown. Each lane represents a different cell line. Aberrant E-cadherin expression is seen in Kato 3, consistent with a previously described mutation. AGS expressed very low levels of E-cadherin and no α-catenin. A truncated β-catenin is seen in HSC39 and HSC40A, both derived from the same tumour. γ-Catenin is normally expressed in all cell lines. A truncated APC protein product is seen in HT29 and no APC is expressed by Kato 3

Cell morphology and phenotype was assessed using phase contrast microscopy, under standard cell culture conditions. Kato 3 cells grow as single cells or small cell aggregates floating in suspension with a few scattered round cells adhering to the plastic. HSC39 and HSC40a also grow in vitro as a suspension of single cells or as loosely associated aggregates with no adherence to plastic, while MKN45 grows as a loosely adherent monolayer with spindle-shaped cells exhibiting poor cell–cell contact. MKN7 and AGS both grow as flat coherent monolayers. Impaired cell–cell adhesion was therefore apparent at phase contrast microscopy of cells in culture, in four of the six gastric cell lines.

 © Cancer Research Campaign 1999

**Table 1** E-cadherin and catenin expression by immunofluoresence staining in gastric carcinoma cell lines

| | E-cadherin | α-catenin | β-catenin | γ-catenin | p120$^{ctn}$ |
|---|---|---|---|---|---|
| Kato 3 | Membranous | Cytoplasmic | Patchy membranous | Patchy membranous | Membranous |
| AGS | Negative | Negative | Cytoplasmic | Cytoplasmic | Membranous |
| MKN7 | Membranous | Patchy membranous | Membranous | Membranous | Membranous |
| MKN45 | Membranous | Membranous | Membranous | Membranous | Membranous |
| HSC39 | Membranous | Cytoplasmic | Cytoplasmic | Cytoplasmic | Membranous |
| HSC40a | Membranous | Cytoplasmic | Cytoplasmic | Cytoplasmic | Membranous |

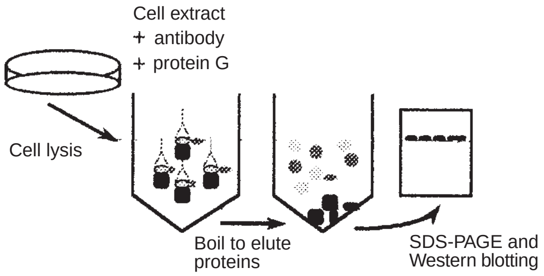

**Figure 2** Schematic representation of the examination of protein–protein interactions through immunoprecipitation followed by Western blotting. Equal aliquots of cell extract are each mixed with the immunoprecipitating antibody. Immune complexes are formed consisting of the immunoprecipitating antibody, its antigen and any other protein, which binds to primary antigen to form a multimolecular complex. The immunoprecipitates are then captured using protein G beads, which are washed, and the protein components of the complexes eluted into suspension and then separated under reducing conditions by SDS-PAGE and Western blotting. The presence of interacting proteins can then be detected by probing the nitrocellulose blots with a panel of antibodies to components of the cadherin–catenin complex

Functional assessment of calcium-dependent cell–cell adhesion was performed using a cell–cell aggregation assay as described by Takeichi et al (1997) with modifications. In the presence of a normally functioning cadherin–catenin complex as seen in MDCK and HT29 there was a progressive fall in the number of single cells with time (65% at 1 h). This is demonstrated by a fall in aggregation index to 0.35 at 90 min in calcium-containing medium. The aggregation index is calculated as the number of single cells at time × number of single cells at time 0. Cadherin-dependent cell–cell adhesion in HT29 is abolished by incubation of cells in calcium-free media, with no change in the aggregation index occurring with time. No calcium-dependent cell–cell aggregation could be demonstrated, in any of the gastric carcinoma cell lines suggesting impairment of E-cadherin-mediated intercellular adhesion in these cell lines.

### Expression of E-cadherin and the catenins in gastric carcinoma cell lines

Aberrant expression of E-cadherin and/or catenins was demonstrated in a number of the cell lines examined. Western blot analysis (Figure 1) revealed very low levels of E-cadherin in AGS, which were often barely discernible in repeat experiments. Aberrant E-cadherin expression was detected in Kato 3 and MKN45. Two protein bands reactive for E-cadherin were detected in Kato 3, one at 120 kDa, and a second at a 130 kDa. MKN45 demonstrated one band at the expected molecular weight of 120 kDa and the second at 80 kDa, which is likely to represent the extracellular tryptic product of E-cadherin as previously described (Frixen et al, 1991). E-cadherin was detected at the expected molecular weight of 120 kDa in HSC39, HSC40A and MKN7, although levels of E-cadherin expression were lower in MKN7 compared to control (HT29) and other gastric carcinoma cell lines.

α-catenin expression was absent in AGS, but expressed at the expected molecular weight of 102 kDa in the remaining five cell lines, although levels were reduced in three of these cell lines, Kato 3, HSC39 and HSC40A, compared to control. β-catenin was truncated at 80 kDa in HSC39 and HSC40A, as previously described by the originators (Kawanishi et al, 1995). Finally, all six cell lines expressed γ-catenin and p120$^{ctn}$ (data not shown) in comparable amounts and at the expected molecular weight. A band at 300 kDa immunoreactive for APC was detected in all but one cell line, Kato 3, which was negative for APC, suggesting the probable presence of a mutation of the *APC* gene. A truncated APC protein migrating at 200 kDa was detected in HT29, confirming a previously described mutation.

### Cellular localization of E-cadherin and the catenins in gastric carcinoma cell lines

Staining results are summarized in Table 1. Immunofluoresence staining in the control cell line, HT29, revealed co-localization of E-cadherin and the catenins at the cell membrane, with increased staining intensity at points of cell–cell contact consistent with adherens junctions. Diffuse cytoplasmic and nuclear staining was additionally observed with β-catenin, reflecting its now known role as transcriptonal regulator.

E-cadherin membranous staining was absent in AGS, and patchy with low intensity in MKN7. These findings agree with Western blotting data showing absent and reduced levels of E-cadherin in these cell lines respectively. Linear membranous staining was present, however, in the remaining four gastric carcinoma cell lines, including Kato 3 and MKN45, which had aberrant E-cadherin expression on Western blotting. Loss of membranous staining for α-catenin was seen in Kato 3 and HSC39, HSC40a and AGS. There was patchy membranous staining for β- and γ-catenin in Kato 3, while staining was cytoplasmic in HSC39, HSC40a and AGS. p120$^{ctn}$ stained in a membranous distribution in all cell lines. Loss of membranous localization of either E-cadherin or the catenins, implies corresponding loss of their adhesive function, though disruption of adherens junctions.

Staining for the EGFR revealed intense membranous distribution in Kato 3, and faint membranous staining with a punctate pattern in MKN7 and MKN45. It was absent in AGS, HSC39 and HSC40a.

© Cancer Research Campaign 1999 

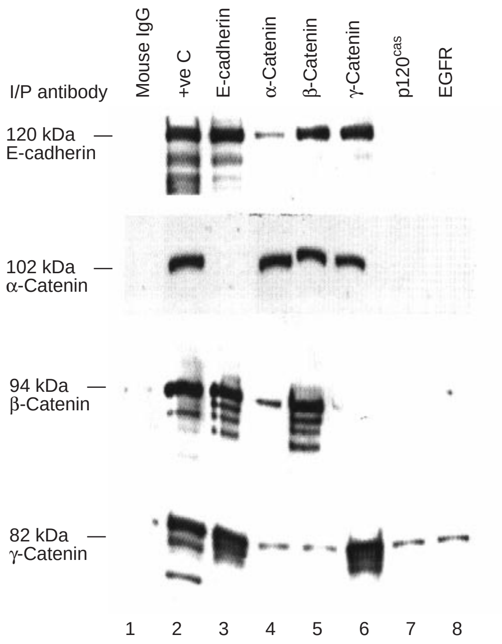

**Figure 3** Western blotting of cell extracts after immunoprecipitation using E-cadherin, catenin and EGFR antibodies in HT29. This Figure shows results of sequential immunoprecipitation (I/P) of cell extracts, followed by Western blotting to examine E-cadherin–catenin complex composition in HT29. Each lane represents the results of immunoprecipitation with one antibody, indicated above the panels. Precipitated proteins were separated by SDS-PAGE and subjected to Western blotting using the antibody indicated to the right of the diagram. E-cadherin was co-precipitated with β- and γ-catenin, and to a lesser extent with α-catenin antibodies (top panel, lanes 4–6). α-Catenin is seen to bind to β-catenin and γ-catenin (second panel, lanes 5–6), while both β- and γ-catenin were immunoprecipitated in small quantities by EGFR antibodies (bottom panel, lane 8). Lane 1 is a negative control I/P with mouse IgG, while lane 2 is a positive control I/P using the same antibody as the Western blot

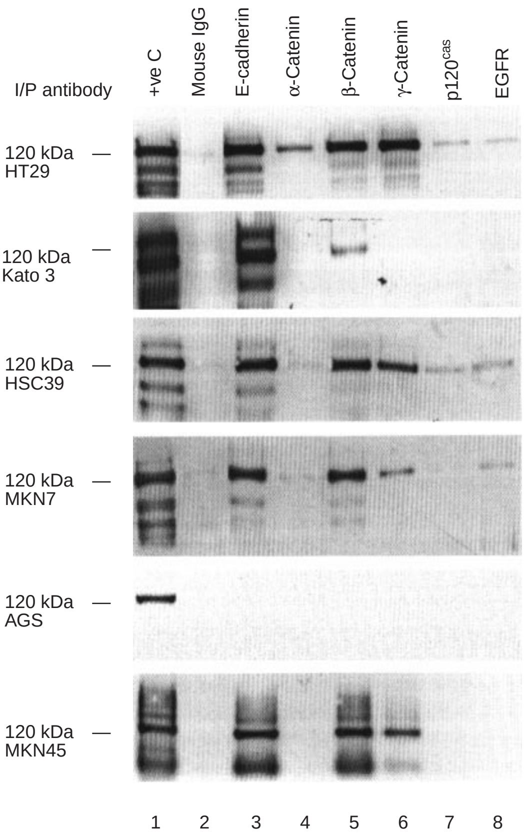

**Figure 4** E-cadherin Western blots of gastric cell line extracts after sequential immunoprecipitation with E-cadherin, catenin and EGFR antibodies. Cell extracts indicated to the right were immunoprecipitated with antibodies to E-cadherin, α- β- and γ-catenin, p120$^{ctn}$, and EGFR as indicated above. Precipitated proteins were separated by SDS-PAGE and subjected to Western blotting for the presence of E-cadherin in the immunoprecipitates. E-cadherin binding to β-catenin was significantly reduced in Kato 3 (second panel, lane 5) relative to the total amount of E-cadherin precipitated by E-cadherin antibodies (lane 3), and no E-cadherin was detectable in γ-catenin immunoprecipitates in this cell line (second panel, lane 6). E-cadherin binding to p120$^{ctn}$ could not be demonstrated in Kato 3, MKN7 and MKN45 (lane 7). AGS fails to express E-cadherin, so as expected no proteins could be demonstrated in the immunoprecipitates (panel 5). Lane 1 is a positive control I/P with β-catenin antibodies, while lane 2 is a negative control I/P with mouse IgG

### Protein interactions within the cadherin–catenin complex in gastric carcinoma cell lines

We examined protein interactions within the cadherin–catenin complex, using sequential immunoprecipitation and immunoblotting with E-cadherin, α-, β-, γ-catenin, p120$^{ctn}$, EGFR and APC antibodies. The protein concentration of cell extracts was measured by BioRad protein assay and equal aliquots were each mixed with the immunoprecipitating antibody. Immune complexes are formed consisting of the immunoprecipitating antibody, its antigen and any other protein, which binds to primary antigen to form a multimolecular complex. The immunoprecipitates are then captured using protein G beads, which are washed, and the protein components of the complexes eluted into suspension and then separated under reducing conditions by SDS-PAGE and Western blotting. The presence of interacting proteins can then be detected by probing the nitrocellulose blots with a panel of antibodies to components of the cadherin catenin complex (see diagramatic representation, Figure 2).

HT29 was used to demonstrate the normal composition of the cadherin–catenin complex (Figure 3). The figure consists of four Western blots for E-cadherin, α-, β- and γ-catenin performed on HT29 cell extracts after immunoprecipitation with the antibody shown above the panel. E-cadherin was co-immunoprecipitated with β- and γ-catenin and, to a lesser extent, with α-catenin antibodies suggesting the presence of a binding interaction between these proteins (top panel). α-catenin was precipitated with both β-catenin and γ-catenin (second panel), while both β and γ-catenin were immunoprecipitated in small quantities by EGFR antibodies. These findings are in agreement with published data suggesting that E-cadherin forms a complex with β- or γ-catenin which in turn bind to α-catenin (Ozawa and Kember, 1992). p120$^{ctn}$ has been shown to bind directly to E-cadherin (Reynolds, 1994), although in our hands, this interaction appeared to be highly sensitive to lysis and wash conditions, and only demonstrable when using very mild non-ionic conditions. EGFR has been shown to interact directly with β- and γ-catenin (Hoschuetzky et al, 1994). In each experiment a negative control immunoprecipitation with non-immune mouse IgG was performed.

Abnormalities of complex composition were demonstrated in a number of gastric cell lines. Kato 3, which is known to have an E-cadherin mutation, demonstrated significantly reduced binding of E-cadherin to β-catenin compared to other cell lines (Figure 4, lane 5). Lane 3 represents the total amount of E-cadherin precipitated

 © Cancer Research Campaign 1999

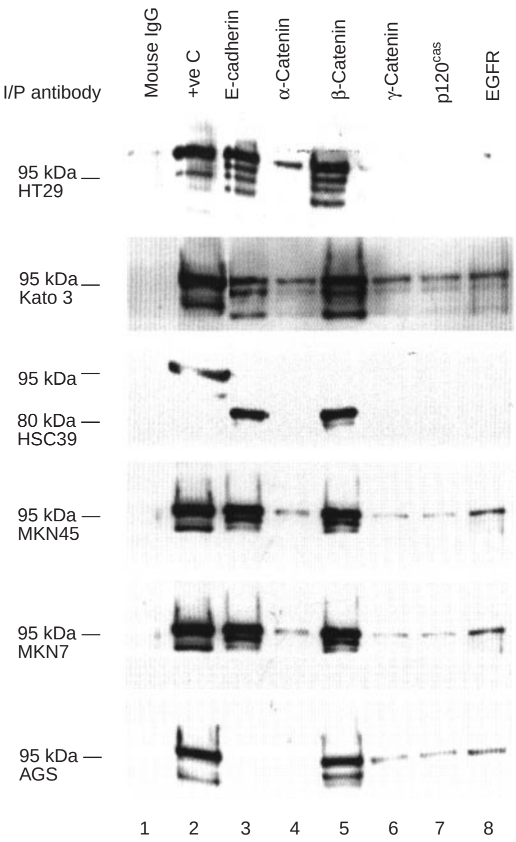

**Figure 5** β-catenin Western blots of gastric cell line extracts after sequential immunoprecipitation with E-cadherin, catenin and EGFR antibodies. Cell extracts from six cell lines indicated to the right were immunoprecipitated with antibodies to E-cadherin, α-, β- and γ-catenin, p120*cas* and EGFR as indicated above the panels. Precipitated proteins were separated by SDS-PAGE and subjected to Western blotting for the presence of β-catenin in the immunoprecipitates. β-Catenin was immunoprecipitated with E-cadherin in all cell lines apart from AGS, which fails to express the E-cadherin protein (lane 5). The truncated β-catenin protein (80 kDa) in HSC39 fails to immunoprecipitate with α-catenin or EGFR (third panel, lanes 4 and 8). Lane 1 is a negative control I/P using mouse IgG, while lane 2 is a positive control I/P using β-catenin antibodies

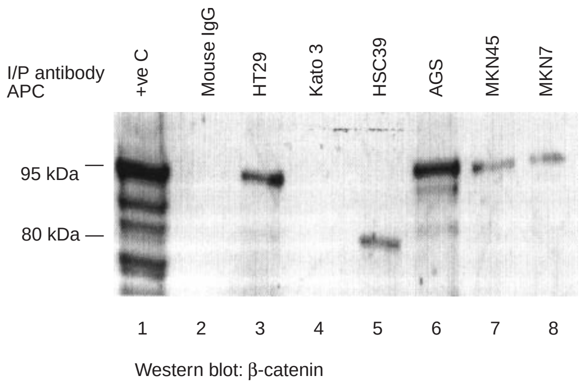

**Figure 6** β-catenin Western blot of gastric cell line extracts after immunoprecipitation with APC antibodies. Cell extracts from six cell lines indicated above the panel were immunoprecipitated with antibodies to APC (Ab28). Precipitated proteins were separated by SDS-PAGE and subjected to Western blotting for the presence of β-catenin in the immunoprecipitates. β-Catenin was precipitated by APC antibodies in all cell lines except Kato 3, which does not express APC protein (lane 4). Lane 1 is positive control immunoprecipitation (I/P) with β-catenin antibody, while lane 2 is a negative control I/P with mouse IgG

by E-cadherin antibodies in a parallel experiment performed simultaneously. In other words, only a small proportion of the total amount of E-cadherin (lane 3), could be precipitated with β-catenin antibodies (lane 5). This can be compared to the equal E-cadherin band densities arising from immunoprecipitation with E-cadherin or β-catenin antibodies in the other cell lines, suggesting that the majority of the β-catenin in these cell lines is E-cadherin bound. Equal amounts of cell extract protein, and immunoprecipitating antibody were used in each experiment, making a comparison of band density for a particular protein between cell lines possible. AGS, which is negative for E-cadherin on Western blotting, showed no E-cadherin protein on immunoprecipitation with E-cadherin or catenin antibodies. E-cadherin binding to p120*ctn* also appeared to be disrupted in Kato 3, MKN7 and MKN45 (Figure 4, lane7). E-cadherin was present in EGFR immunoprecipitates in three cell lines, HT29, HSC39 and MKN7. This is an unexpected finding and may represent an indirect interaction of E-cadherin with EGFR, through β-catenin, due to precipitation of higher order complexes.

β-catenin was immunoprecipitated by E-cadherin suggesting preservation of the β-catenin–E-cadherin interaction antibodies in all cell lines, except AGS, which fails to express E-cadherin (Figure 5, lane 3). There was associated loss of α/β-catenin co-precipitation in AGS suggesting loss of this interaction in the absence of E-cadherin (Figure 5, lane 4, bottom panel). The truncated β-catenin protein in HSC39 failed to co-precipitate with α-catenin or EGFR, implying loss of the corresponding binding site (Figure 5, lane 7 and 8, third panel). The β-catenin–EGFR interaction was preserved in all other cell lines.

In some of our immunoprecipitation experiments separation of β-catenin and γ-catenin into two distinct complexes was apparently not complete, with β-catenin and γ-catenin appearing in the reciprocal immunoprecipitations (Figure 5, lane 6). In addition, β-catenin was immunoprecipitated with p120*ctn* antibodies immunoprecipitates (Figure 5, lane 7), and E-cadherin with EGFR (Figure 4, lane 8). These unexpected interactions may reflect co-immunoprecipitation of a minor subpopulation of 'higher order complexes'.

On immunoprecipitation with APC antibodies, β-catenin was seen to be present in the immunoprecipitates in all cell lines apart from Kato 3, which we have shown to have loss of APC expression (Figure 6). This suggests preservation of the APC–β-catenin interaction in these cell lines even in the presence of a truncated β-catenin protein as in HSC39.

### DISCUSSION

Loss of cadherin-mediated intercellular adhesion is an important contributory mechanism in tumour pathogenesis. It is postulated to remove contact inhibition of proliferation, thus allowing escape from growth control signals (St Croix et al, 1998). Loss of cadherin-mediated adhesion may also act by potentiating tumour cell detachment from the primary site, and resulting in dissemination of malignant cells to form metastases at distant sites (Birchmeier et al, 1993). E-cadherin is thus postulated to act both as a growth suppressor and invasion suppressor. An intact cadherin–catenin complex is required, however, for maintenance of normal intercellular adhesion, with mutations of either E-cadherin or α/β-catenins being sufficient to disrupt adherens junction function.

*© Cancer Research Campaign 1999*

In this study, examination of cell morphology, protein expression and localization, and molecular organization of the cadherin–catenin complex in six gastric carcinoma cell lines have demonstrated evidence of widespread dysfunction of calcium-dependent intercellular adhesive mechanisms. E-cadherin is the principal mediator of intercellular adhesion, and adherens junction integrity in epithelial cells. When it functions normally, disruption of other intercellular adhesion molecules has little effect on adhesive function. Four of the six cell lines tested showed morphological evidence of reduced intercellular adhesion, while all failed to demonstrate any evidence of calcium-dependent intercellular adhesion when examined in a cell–cell aggregation assay.

A variety of mutations in the cadherin–catenin system have previously been described in a number of these cell lines. We confirm these defects, and additionally demonstrate loss of expression of some components of the complex. By a detailed examination of protein expression, cellular localization by immunocytochemisty, and co-immunoprecipitation analysis to assess protein–protein interactions within the cadherin–catenin complex, we have identified putative defects in the corresponding adhesion systems.

Kato III is known to have an E-cadherin point mutation, resulting in the production of aberrant mRNA, which is manifested by the presence of two E-cadherin bands on Western blotting, at 120 and 130 kDa (Oda et al, 1994). E-cadherin dysfunction in Kato III secondary to mutation is demonstrable by its tendency to grow as free-floating cells with little tendency towards calcium-dependent cell–cell aggregation. Immunoprecipitation experiments revealed reduced E-cadherin binding to β-catenin and no binding to γ-catenin, suggesting disruption of cadherin–catenin complex integrity. These abnormalities were associated with reduced intensity and heterogeneity of staining for the catenins at cell borders. It seems likely that the E-cadherin mutation results either in loss of the catenin-binding site or steric changes interfering with binding to the catenins, and hence loss of catenin localization to the adherens junction and interruption of the adherens junction link to the actin cytoskeleton.

An E-cadherin mutation has also been described in MKN45 (Oda et al, 1994), in this case at the extracellular calcium-binding domain. This would explain the loss of cell–cell aggregation despite the continued normal expression of both E-cadherin and catenins at the cell membrane and the normal protein interactions with the catenins. The lower molecular weight band in the E-cadherin blot thought to represent the extracellular tryptic product of E-cadherin as previously described (Frixen et al, 1991).

MKN7 also demonstrated total loss of cell–cell aggregation despite expression of all components of the cadherin–catenin complex at the expected molecular weights and normal membranous localization. E-cadherin levels were low compared to other cell lines and to control, both on Western blotting and immunocytochemical staining. On immunoprecipitation with E-cadherin antibodies, the only aberration in protein interactions was failure to demonstrate the expected binding of p120*ctn* to E-cadherin, the significance of which is uncertain. The explanation for impaired intercellular adhesion is less obvious in this cell line. A point mutation in the E-cadherin, or catenin genes has not been excluded.

HSC39 has previously been shown to have a β-catenin mutation resulting in protein truncation with loss of the α-catenin-binding site (Kawanishi et al, 1995). This is demonstrated by loss of the E-cadherin–β-catenin interaction with α-catenin (Figure 5, lane 4, panel 3), which in turn links it to the actin cytoskeleton. Immunofluoresence staining demonstrated diffuse cytoplasmic staining of the catenins with loss of membranous localization, suggesting failure to associate with E-cadherin at the cell membrane. These abnormalities in the cadherin–catenin complex are reflected by loss of calcium-dependent cadherin-mediated cell–cell adhesion, and by failure of cells to form a monolayer in culture.

AGS showed failure of E-cadherin and α-catenin on Western blotting and immunocytochemical staining. Loss of α-catenin expression may be secondary to loss of E-cadherin. Previous data have suggested that E-cadherin confers stability on α-catenin, in the absence of which it is broken down in the cytoplasm. Normal β-catenin expression was seen on Western blotting, but this was totally cytoplasmic in distribution. This is not surprising as its location at the cell membrane depends on binding to E-cadherin, which in the case of AGS fails to be expressed.

p120*ctn* binds to the juxtamembranous region of the E-cadherin cytoplasmic tail, which has been shown to support the lateral clustering of E-cadherin molecules at the cell surface. p120*ctn* is thought to act as an intermediary binding protein allowing clustering of E-cadherin at the cell surface, a function which allows modulation of adhesive strength under different cirmumstances (Yap et al, 1998). We found the E-cadherin–p120*ctn* interaction to be highly sensitive to lysis and buffer conditions, with easy disruption of the complex. The continued membranous localization of p120*ctn* in all the cell lines, suggests maintenance of its normal binding relationship with E-cadherin, as its membranous localization depends on this relationship.

Apart from binding to E-cadherin at the cell membrane, a significant proportion of the catenin pool is free cytoplasmic protein, or exists in the form of cadherin-independent α/β-catenin, or α/γ-catenin complexes. These complexes are thought to influence the titration of catenins between cadherin and APC complexes. Mutagenesis studies have shown that for tumour formation to ensue, truncation of the APC protein must include loss of that part of the protein responsible for the ability to down-regulate β-catenin. The resulting increase in cytoplasmic β-catenin levels, and the ensuing increased translocation to the nucleus, results in autonomous transcription of Tcf target genes, known to be specific to colonic epithelium, and thus contributes to neoplastic transformation.

Mutations of the amino terminal regulatory domain of β-catenin have been demonstrated in 48% of colorectal tumours lacking APC mutations (Sparks et al, 1998). Mutations in this area of the protein render it insensitive to APC-mediated degradation. β-catenin mutations were also present in a similar proportion of colorectal adenomas suggesting that like APC, these mutations arise early in tumorigenesis. In an exciting recent discovery, one of the cancer-promoting genes at the end of the β-catenin signal transduction pathway has been shown to be the oncogene c-*myc* (Sparks, et al, 1998). Wild-type APC is thought to prevent β-catenin from forming a complex with Tcf-4 and activating c-*myc* expression. But when APC is mutated, β-catenin accumulates and is translocated to the nucleus where it results in sustained transcriptional activation of certain genes, including c-*myc*, and causes tumour growth. This is a rare example of loss of a tumour suppressor gene resulting in activation of an oncogene. The *APC* gene is mutated in up to 20% of gastric carcinomas and in a similar proportion of gastric adenomas (Horii et al, 1992; Tamura et al, 1994). The data presented in this study demonstrate preservation of the APC–β-catenin interaction in 5 gastric carcinoma cell lines even in the presence of a truncated β-catenin protein as in HSC39.

© Cancer Research Campaign 1999

In conclusion, we have demonstrated frequent abnormalities of expression and function of the cadherin–catenin complex, correlated with evidence of impaired protein–protein interactions within the cadherin–catenin complex. These abnormalities manifest with disturbance of cadherin-mediated intercellular adhesion in gastric carcinoma cell lines. While the defects demonstrated were heterogeneous, affecting E-cadherin, α and β-catenin, the functional result was similar, that of loss of cadherin-mediated cell–cell adhesion. These findings support previously reported in vivo findings of a role for inactivation of E-cadherin-mediated intercellular adhesion in the pathogenesis and progression of gastric carcinoma.

### REFERENCES

Becker KF, Atkinson MJ, Reich U, Huang HH, Nekarda H, Siewert BR and Hofler H (1993) Exon skipping in the E-cadherin gene transcript in metastatic human gastric carcinomas. *Hum Mol Genet* **2**: 803–804

Becker KF, Atkinson MJ, Reich U, Becker I, Nekarda H, Siewert JR and Hofler H (1994) E-cadherin gene mutations provide clues to diffuse type gastric carcinomas. *Cancer Res* **54**: 3845–3852

Behrens J, von Kreis JP, Kuhl M, Bruhn L, Wedlich D, Grosschedl R and Birchmeier W (1996) Functional interaction of β-catenin with the transcription factor LEF-1. *Nature* **382**: 638

Bergerheim USR, Kunimi K, Collins BP and Ekman P (1991) Deletion mapping of chromosomes 8, 10 and 16 in prostate carcinoma. *Genes Chrom Cancer* **3**: 215–220

Birchmeier W, Michael Weidner K and Behrens J (1993) Molecular mechanisms leading to loss of differentiation and gain of invasiveness in epithelial cells. *J Cell Sci*. **17**: 159–164.

Bringuier PP, Umbas R, Schaafsma HE, Karthaus HF, Debruyne FM and Schalken JA (1993) Decreased E-cadherin immunoreactivity correlates with poor survival in patients with bladder tumours. *Cancer Res* **53**: 3241–3245

Carter BS, Ewing CM, Ward WS, Treiger BF, Aalders TW, Schalken JA, Epstein JI, and Issac WB (1991) Allelic loss of chromosomes 16q and 10q in human prostate cancer. *Proc Natl Acad Sci USA* **87**: 8751–8755

Downing JR and Reynolds AB (1991) PDGF, CSF-1, and EGF induce tyrosine phosporylation of p120, a pp60src transformation associated substrate. *Oncogene* **6**: 607–613

Efstathiou JA, Noda M, Rowan A, Dixon C, Chinery R, Jawhari A, Hattori T, Wright NA, Bodmer W, and Pignatlli M (1998) Intestinal trefoil factor controls the expression of the adenomatous polyposis coli-catenin and the E-cadherin–catenin complexes in human colon carcinoma cells. *Proc Natl Acad Sci USA* **95**: 3122–3127

Frixen UH, Behrens J, Sachs M, Eberle G, Voss B, Warda A, Locner D and Birchmeier W (1991) E-cadherin-mediated cell–cell adhesion prevents invasiveness of human carcinoma cells. *J Cell Biol* **113**: 173–185.

Geiger B, Tokuyasu KT, Dutton AH and Singer SJ (1980) Vinculin, an intracellular protein localized at specialized sites where microfilament bundles terminate at cell membranes. *Proc Nat Acad Sci USA* **7777**: 4127–4131

Guilford P, Hopkin J, Harraway J, McLeod M, McLeod N, Harawira P, Taite H, Scoular R, Miller A and Reeve AE (1998) E-cadherin germline mutations in familial gastric carcinoma. *Nature* **392**: 402–404

Gumbiner B (1995) Signal transduction by β-catenin. *Curr Opin Cell Biol* **7**: 634–640

Hirano S, Kimoto N, Shimoyama Y, Hirohashi S and Takeichi M (1992) Identification of a neural α-catenin as a key regulator of cadherin function and multicellular organization. *Cell* **70**: 293–301

Horii A, Nakatsuru S, Miyoshi Y, Ichii S, Nagase H, Kato Y, Yanagisawa A, and Nakamura Y (1992) The APC gene, responsible for familial adenomatous polyposis, is mutated in human gastric cancer. *Cancer Res* **52**: 3231–3233

Hoschuetzky H, Aberle H and Kemler R (1994) β-catenin mediates the interaction of the cadherin–catenin complex with epidermal growth factor receptor. *J Cell Biol* **127**: 1375–1380

Jawhari A, Poole S, Jordan S, Browne P, Pignatelli M and Farthing MJG (1997) Abnormal immunoreactivity of the E-cadherin–catenin complex in gastric carcinoma: relationship with patient survival. *Gastroenterology* **112**: 46–54

Kawanishi J, Kato J, Sasaki K, Fujii S, Watanabe N and Niitsu Y (1995) Loss of E-cadherin-dependent cell–cell adhesion due to mutation of the β-catenin gene in a human cancer cell line, HSC-39. *Mol Cell Biol* **15**: 1175–1181

Korinek V, Barker N, Morin PJ, van Wichen D, de Weger R, Kinzler KW, Vogelstein B and Clevers H (1997) Constitutive transcriptional activation by a β-catenin–Tcf complex in APC−/−colon carcinoma. *Science* **275**: 1784–1787

Laemmli UK (1970) Cleavage of structural proteins during the assembly of the head of bacteriophage T4. *Nature* **227**: 680–685

Lemoine NR, Jain S, Silvestre F, Lopes C, Hughes CM, McLelland E, Gullick WJ and Filipe MI (1991) Amplification and over-expression of the EGF receptor and c-erbB-2 proto-oncogenes in human stromach cancer. *Br J Cancer* **64**: 79–83

Matsuura K, Kawanishi J, Fujii S, Imamura M, Hirano S, Takeichi M and Niitsu Y (1992) Altered expression of E-cadherin in gastric cancer tissues and carcinomatous fluid. *Br J Cancer* **66**: 1122–1130

Munemitsu S, Albert I, Souza B, Rubinfeld B and Polakis P (1995) Regulation of intracellular β-catenin levels by the adenomatous polyposis coli (APC) tumour-suppressor protein. *Proc Natl Acad Sci USA* **92**: 3046–3050

Nagafuchi A and M Takeichi (1988) Cell-binding function of E-cadherin is regulated by the cytoplasmic domain. *EMBO J* **7**: 3679–3684

Nagafuchi A and Takeichi M (1989) Transmembrane control of cadherin-mediated cell adhesion: a 94 kD protein functionally associated with a specific region of the cytoplasmic domain of E-cadherin. *Cell Regul* **1**: 37–44

Nigam AK, Savage FJ, Boulos PB, Stamp GW, Liu D and Pignatelli M (1993) Loss of cell–cell and cell–matrix adhesion molecules in colorectal cancern. *Br J Cancer* **68**: 507–514

Ochiai A, Akimoto S, Kanai Y, Shibata T, Oyama T, and Hirohashi S (1994) c-erbB-2 gene product associates with catenins in human cancer cells. *Biochem Biophys Res Commun* **205**: 73–78

Oda T, Kanai Y, Oyama T, Yoshiura K, Shimoyama Y, Birchmeier W, Sugimura T and Hirohashi S (1994) E-cadherin gene mutations in human gastric carcinoma cell lines. *Proc Natl Acad Sci USA* **91**: 1958–1962

Oka H, Shiozaki H, Kobayashi K, Inoue M, Tahara H, Kobayashi T, Takatsuka Y, Matsuyoshi N, Hirano S and Takeichi M (1993) Expression of E-cadherin cell adhesion molecules in human breast cancer tissues and its relationship to metastasis. *Cancer Res* **53**: 1696–1701

Ozawa M and Kemler R (1992) Molecular organization of the uvomorulin–catenin complex. *J Cell Biol* **116**: 989–996

Peifer M (1995) Cell adhesion and signal transduction: the armadillo connection. *Trend Cell Biol* **5**: 224–229

Pignatelli M, Ansari TW, Gunter P, Liu D, Hirano S, Takeichi M, Kloppel G and Lemoine NR (1994) Loss of membranous E-cadherin expression in pancreatic cancer: correlation with lymph node metastasis, high grade and advanced stage. *J Pathol* **174**: 243–248

Rubinfeld B, Souza B, Albert I, Muller O, Chamberlain SH, Masiarz FR, Munemitsu S and Paulakis P (1993) Association of the APC gene product with β-catenin. *Science* **262**: 1731–1734

Sato T, Tanigami A, Yamakawa K, Akiyama F, Dasumi F, Sakamato G and Nakamura Y (1990) Allotype of breast cancer: cumulative allele losses promote tumor progression in primary breast cancer. *Cancer Res* **50**: 7184–7189

Shapiro L, Fannon AM, Kwong PD, Thompson A, Lehmann ML, Grubel G, Legrand JF, Als-Nielson J, Coleman KR and Hendrickson WA (1995) Structural basis of cell-cell adhesion by cadherins. *Nature* **374**: 327–386

Shibamoto S, Haykawa M, Takeuchi K, Hori T, Oku N, Miyazazwa K, Kitamura N, Takeichi M and Ito F (1994) Tyrosine phosphorylation of β-catenin and plakoglobin enhanced by hepatocyte growth factor and epidermal growth factor in human carcinoma cells. *Cell Adhes Commun* **1**: 295–305

Shimoyama Y and Hirohashi S (1991) Expression of E- and P-cadherin in gastric carcinomas. *Cancer Res* **51**: 2185–2192

Shiozaki H, Tahara H, Oka H, Miyata M, Kobayashi K, Tamura S, Iihara K, Doki Y Hirano S, Takeichi M et al. (1991) Expression of immunoreactive E-cadherin adhesion moleules in human cancers. *Am J Pathol* **139**: 17–23

Shiozaki H, Lihara K, Oka H, Kadowaki T, Matsui S, Gofuku J, Inoue M, Nagafuchi A, Tsukita S and Mori T (1994) Immunohistochemical detection of α-catenin expression in human cancers. *Am J Pathol* **144**: 667–674

Sparks AB, Morin PJ, Vogelstein B and Kinzler KW (1998) Mutational analysis of the APC/β-catenin pathway in colorectal cancer. *Cancer Res* **58**: 1130–1134

Staddon J, Smales C, Schulze C, Esch FS, and Rubin LL (1995) p120, a p120-related protein (p100), and the cadherin/catenin complex. *J Cell Biol* **130**: 369–381

St Croix B, Sheehan C, Rak JW, Florenes VA, Slingerland JM and Kerbel RS (1998) E-cadherin dependent growth suppression is mediated by the cyclin-dependent kinase inhibitor $p27^{KIP1}$. *J Cell Biol* **142**: 557–571

Su LK, Vogelstein B and Kinzler KW (1993) Association of the APC tumour suppressor protein with catenins. *Science* **262**: 1734–1737

Tahara E (1995) Molecular biology of gastric cancer. *World J Surg* **19**: 484–490

© Cancer Research Campaign 1999 

Takeichi M (1977) Functional correlation between cell adhesive properties and some cell surface proteins. *J Cell Biol* **75**: 464–474

Takeichi M (1991) Cadherin cell adhesion receptors as morphogenetic regulators. *Science* **251**: 1451–1455

Tamura G, Maesawa C, Suzuki Y, Tamada H, Satoh M, Ogasawara S, Kashiwaba M and Satodate R (1994) Mutations of the APC gene occur during early stages of gastric adenoma development. *Cancer Res* **54**: 1149–1115

Umbas R, Schalken JA, Aalders TW, Carter BSm Karthaus HF, Schaafsma HE, Debruyue FM and Isaacs WB (1992) Expression of cellular adhesion molecule E-cadherin is reduced or absent in high grade prostate cancer. *Cancer Res* **52**: 5104–5109

Volberg T, Zick Y, Dror R, Sabanay I, Gilon C, Levtzki A and Geiger B (1992) The effect of tyrosine-specific protein phosphorylation in the assembly of adherens-type junctions. *EMBO J* **11**: 1733–1742

Wilding J, Vousden KH, Soutter P, McCrea PD, Del Buono R and Pignatelli M (1996) E-cadherin transfection down-regulates the epidermal growth factor receptor and reverses the invasiv phenotype of human papilloma virus transfected keratinocytes. *Cancer Res* **56**: 5285–5292

Yap AS, Niessen CM and Gumbiner BM (1998) The juxtamembrane region of the cadherin cytoplasmic tail supports lateral clustering, adhesive strengthening, and interaction with p120ctn. *J Cell Biol* **141**: 779–789

 *© Cancer Research Campaign 1999*
